# What does germane load mean? An empirical contribution to the cognitive load theory

**DOI:** 10.3389/fpsyg.2014.01099

**Published:** 2014-10-01

**Authors:** Nicolas Debue, Cécile van de Leemput

**Affiliations:** ^1^Faculty of Psychological Science and Education, Research Center for Work and Consumer Psychology, Université Libre de BruxellesBrussels, Belgium; ^2^National Fund for Scientific Research (FRS-FNRS)Brussels, Belgium

**Keywords:** cognitive load measurement, eye-tracking, objective measures, online newspaper, information retention

## Abstract

While over the last decades, much attention has been paid to the mental workload in the field of human computer interactions, there is still a lack of consensus concerning the factors that generate it as well as the measurement methods that could reflect workload variations. Based on the multifactorial Cognitive Load Theory (CLT), our study aims to provide some food for thought about the subjective and objective measurement that can be used to disentangle the intrinsic, extraneous, and germane load. The purpose is to provide insight into the way cognitive load can explain how users' cognitive resources are allocated in the use of hypermedia, such as an online newspaper. A two-phase experiment has been conducted on the information retention from online news stories. Phase 1 (92 participants) examined the influence of multimedia content on performance as well as the relationships between cognitive loads and cognitive absorption. In Phase 2 (36 participants), eye-tracking data were collected in order to provide reliable and objective measures. Results confirmed that performance in information retention was impacted by the presence of multimedia content such as animations and pictures. The higher number of fixations on these animations suggests that users' attention could have been attracted by them. Results showed the expected opposite relationships between germane and extraneous load, a positive association between germane load and cognitive absorption and a non-linear association between intrinsic and germane load. The trends based on eye-tracking data analysis provide some interesting findings about the relationship between longer fixations, shorter saccades and cognitive load. Some issues are raised about the respective contribution of mean pupil diameter and Index of Cognitive Activity.

## Introduction

In parallel with the development of computer science and information technology over the last decades, the workplace has evolved to be ever more mediatized by computers. Subsequently, understanding the constraints that the use of hypermedia represents for the worker has become an ever more important issue in the field of human-computer interaction and technology acceptance. In particular, the study of mental workload has been the subject of close attention because it is often seen as a constraint for the user. In spite of this growing interest, there are still many issues concerning the components of cognitive load (CL) as well as the measurement methods that are currently available. In order to impart a better understanding of these issues, this paper approaches cognitive load as a multifactorial concept, such as is defined by Cognitive Load Theory (CLT) (Sweller, 1988; Sweller et al., 1998). In an attempt to investigate how cognitive load factors are affected by the environmental characteristics of hypermedia, an exploratory study was conducted in the context of online news reading. Firstly, we will be presenting the background of CLT and the cognitive load factors that pertain to this theory as well as the issues related to these concepts. Then, we will focus on the objective and subjective measurement methods that are commonly used to assess cognitive load and our research questions will be stated. As this study involves a two-phase experiment, methods and results will be split and presented accordingly. Finally, results will be discussed, and the limitations and implications of this study will be outlined.

### Cognitive load theory

Based on Hitch and Baddeley's model of working memory (1976), the CLT was originally developed by Sweller (1988) in the fields of education and instructional design. CLT assumes that storage and information processing are based on two interdependent systems. The working memory, that deals with information processing and the long-term memory that stores information in the form of schemata. CLT refers to schema theory (Chi et al., 1981) to explain how individuals acquire and store information by integrating lower-order schemata with higher ones. CLT assumes that working memory resources are limited and that processing and maintaining information uses a certain proportion of these resources. So, once a determined amount of information is stored in a single schema, it can be maintained in working memory at a lower cognitive cost. CLT approaches the cognitive load as a multifactorial concept that encompasses different sources of cognitive load generating specific loads.

Sweller (1988) distinguishes between three different cognitive loads. Intrinsic load (IL) is directly related to the learning material (or task) and defined by the number and interactivity of elements that have to be processed. An element being defined as “anything that needs to be or has been learned, such as concept or procedure” (Sweller, 2010, p. 124). Interactivity concerns the relationships among elements. For instance, a list of chemical symbols could be seen as low interactive material because these elements are not dependent, though learning to solve second degree equations represents a high interactivity material. Intrinsic load is dependent on the learner's level of expertise because, the more experienced he or she is, the more they will be able to shrink information on high-order schemata that minimize the cognitive cost of maintaining elements in working memory. Extraneous Load (EL) refers to those mental resources devoted to elements that do not contribute to learning and schemata acquisition or automation. It is mainly related to the information presentation and the instructional format that could both increase the user's overall cognitive load without enhancing learning. It has to be kept as low as possible in order to keep available an adequate amount of mental resources for learning. Germane load (GL) refers to the mental resources devoted to acquiring and automating schemata in long-term memory. Sweller et al. (1998) conceptualized this load when they observed that some instructional formats could increase cognitive load and learning as well. If EL has to be reduced to avoid exceeding working memory resources, GL must be promoted to enhance learning (Ayres, 2006). With this in mind, CLT can explain how a rise in cognitive load can be beneficial for the task in hand instead of solely being linked to a drop in performance.

Nowadays, there has been a large debate among the CLT community about the triarchic model of cognitive load (see Schnotz and Kürschner, 2007; de Jong, 2010 for a review). Researchers differ on the conceptualization of the loads as well as on the nature of the relationships between them. Schnotz and Kurshner consider that IL refers to task performance while germane load in not related to schemata acquisition but represents others cognitive processes that could enhance learning such as the conscious application of learning strategy. In other words, they claim that learning can occur without germane load but that GL can enhance learning (Schnotz and Kürschner, 2007). Kalyuga (2011) goes a step further and argues that germane and intrinsic loads are two redundant concepts that cannot be distinguished. From his perspective, GL is seen as a theoretical construct without empirical evidence because there is no need to refer to GL to explain the main effects predicated by CLT (e.g., the split attention effect). Furthermore, Galy et al. (2012) provided some empirical evidence to support Schnotz and Kurschner's claim in regard to the fact that GL could be related to strategies set up to enhance learning rather than schemata acquisition. According to de Jong (2010) and Gerjets et al. (2009) one of the main issues concerning these contrasting approaches is the *post-hoc* explanation problem. In the absence of reliable measurements for each load, the CLT cannot ever be refuted because it is always possible to attribute variation in the overall cognitive load to a source that corroborates the initial assumptions. For example, assuming that the overall load is kept constant, a decrease in performance will be attributed to a rise in extraneous load that impairs germane cognitive processes. Conversely, if the performance increases it will be attributed to a germane load enhancement made possible by a drop in extraneous load. In spite of this well-known problem, the CLT community still faces difficulties when it comes to the measurement methods to distinguish between types of load (Kirschner et al., 2011). In this regard, our study aims to provide some food for thought about the subjective and objective methods that can be used to disentangle the three kinds of load. The next section will present the measures that can account for cognitive load variations and how they have been applied with regard to CLT.

### Measurement methods

This section presents the measurement methods used to assess cognitive load with an emphasis on measures designed to distinguish between different loads. There are three types of methods to address the measure of cognitive load: subjective rating, performance-based measures and physiological measures (Brunken et al., 2003; Paas et al., 2003; Chanquoy et al., 2007; Galy et al., 2012).

The subjective rating scales are used to assess the cognitive load by referring to the subject's ability to self-evaluate his cognitive processes and the mental effort he requires for an activity. There are two types of scales: unidimensional scales which measure overall cognitive load like Subjective Cognitive Load Measurement scale (Paas and Van Merriënboer, 1994) and multidimensional scales that focus on the different components of load. Despite their intensive usage to estimate cognitive burden, unidimensional scales can be criticized in regard to CLT because they do not do justice to the multifactorial nature of cognitive load. As yet, it is nevertheless recognized that a single item of difficulty is a good indicator of overall cognitive load (Ayres, 2006). On the other hand, a multidimensional scale such as the NASA Task Load Index (NASA-TLX; Hart and Staveland, 1988) gives a broader evaluation of cognitive load. Initially based on six dimensions (performance, mental effort, frustration, task demand, physical demand, temporal demand) this tool has been adapted to the CLT context in several studies (e.g., Gerjets et al., 2004; Scheiter et al., 2006; Ceggara and Chevalier, 2008) in order to provide a measure of the three kinds of load. Cierniak et al. (2009) developed a three-item scale with a difficulty rating for the learning content, difficulty of the material and concentration during learning, so as to, respectively, assess intrinsic, extraneous, and germane load. In spite of the fact that their approach to germane load using the self-reported measure of concentration met their assumptions regarding CLT, they concluded that their tool was unable to distinguish between intrinsic and extraneous load. More recently, Leppink et al. (2013) have developed a ten-item subjective cognitive load scale with students attending lectures on statistics. In contrast to former studies that strove to differentiate measures of load factors (e.g., Ayres, 2006; DeLeeuw and Mayer, 2008), they used multiple items for each type of load in order to increase the accuracy of their tool. Intrinsic load was evaluated with three items referring to the complexity of the material, extraneous load with three items related to the negative characteristics of information providing during the classes and germane load by the mean of four items that dealt with the contribution of explanations and instructions to the learning. The three-factor solution indicated that their tool was able to distinguish properly between each load. Despite these promising results, regarding the evaluation of cognitive load, there are some limitations inherent to self-reported measures. For instance, subjective scales are often administered after the learning task and, in this way, are not able to express the variations of load over time.

Performance-based methods overcome these limitations by providing measures that can reflect variations encountered during the task. Assuming that learning is hindered when working memory capacity is overloaded; a drop in performance could be attributed to an increase in overall cognitive load. One of the most widely used techniques is the dual-task paradigm, in which researchers evaluate the performance in a secondary task performed in parallel to assess the cognitive load devoted to the main task. Various dual-tasks like response time to auditory stimuli (Chevalier and Kicka, 2006; Ceggara and Chevalier, 2008) or a visual monitoring task (DeLeeuw and Mayer, 2008; Cierniak et al., 2009) have been used to evaluate cognitive load. Despite their better temporal accuracy, performance-based methods are intrusive and can interfere with the experimental situation. Furthermore, they are difficult to apply in “real-life” learning situations outside the laboratory.

The third set of methods used for measuring cognitive load concerns physiological measures. The assumption behind these techniques is that cognitive load variations can be reflected in physiological parameters. Unlike rating scales or dual task techniques, these methods provide a high rate measurement with a high degree of sensitivity (Galy et al., 2012). So far, there is no consensus about which parameters should be monitored to best reflect mental load changes. For instance, Backs (1995) or Mulder and Mulder (1981) have shown that heart rate variability is related to task demand. Relationships between heart rate variability, skin conductance and respiration rate have been associated with mental workload while using a driving simulator (Mehler et al., 2009). Behavioral changes like speech rate, prosodic and linguistic changes (Chen et al., 2011) or tapping pace (Albers, 2011) have been related to cognitive load variations. Moreover, neuroimaging techniques like functional Magnetic Resonance Imaging (Whelan, 2007) or Near Infrared Spectroscopy (Ayaz et al., 2012) have been used to gauge variations in cognitive load.

With the democratization of eye-tracking devices, eye-related measures have become one of the most cost effective methods for monitoring user attention, processing demand and mental workload (for an in-depth review of methods and measures, see Rayner, 1998; Holmqvist et al., 2011). There are three classes of eye information that can be observed to monitor cognitive activity: eye movements (fixations and saccades), eye blinks and pupillary response (Chen and Epps, 2012). Fixation location and number of fixations can assess user-attention by indicating how many attentional resources are utilized between stimuli (Hyönä, 2010). Fixation duration and saccade length are assumed to be measures of processing demands or, put differently, cognitive load. There is strong evidence to show that longer fixation duration and shorter saccades are related to higher cognitive load (Holmqvist et al., 2011). Eye blink related measures are more controversial. While eye blink rate is related to task difficulty (Chen and Epps, 2012), blink latency does not seem correlated with cognitive load (Chen et al., 2011). Pupillary response is one of the most extensively studied measures of cognitive load. Relationships between an increase in cognitive load and pupil diameter have been found in various contexts varying from such as simple cognitive tasks (Backs and Walrath, 1992; VanGerven et al., 2004) to naval simulators (De Greef et al., 2009); driving (Marshall, 2007); e-learning (Liu et al., 2011); e-shopping (DiStasi et al., 2011) and an AI web-based tool (Buettner, 2013). Unlike eye movement or blinking, the pupillary reflex is under control of the autonomous nervous system and cannot be voluntary controlled by the subject, which explains the enthusiasm among researchers for this type of measure. Despite its expanding usage, this index suffers from the fact that it is sensitive to luminance variations (Jainta and Baccino, 2010; DiStasi et al., 2011). Based on pupillary response, the Index of Cognitive Activity (Marshall, 2002) has been designed to overcome interference from luminance variation (see Marshall, 2000 for an in-depth explanation). In short, this index is based on the raw pupil data and a wavelet function analysis is made to distinguish unusual increases in the pupil size. It results in an index ranging from 0 to 1 that reflects the level of cognitive effort experienced by the subject.

This short review seeks to outline the diversity of eye-related measures that can be used to monitor cognitive activity. Some of them have been used in studies carried out within the CLT framework (e.g., Amadieu et al., 2009) but there is still no consensus of how cognitive load factors can be independently measured with eye-tracking methods as well as how they are related to a subjective rating scale (VanGog et al., 2009). As it is claim by Gerjets et al. (2009) or Kirschner et al. (2011), there is a pressing need to improve our knowledge of measures of cognitive load.

### Study purpose, context, and research questions

The aim of this paper is twofold. First, in an attempt to contribute to the understanding of the cognitive load components and the nature of germane load in particular, we undertake to provide both subjective and objective measures of cognitive load factors within the CLT framework. Second, we aim to provide insight into the way CLT can explain how users' cognitive resources are utilized in the use of hypermedia such as an online newspaper. Although CLT has been mainly developed in the field of instructional design, there are, indeed, many studies dealing with the design of hypermedia (Hollender et al., 2010) such as e-learning systems (e.g., Amadieu and Tricot, 2006) or websites (Chevalier and Kicka, 2006) that emphasize the usefulness of CLT in these contexts. In the next section, research related to online news design is briefly introduced and assumptions regarding CLT are presented. Next, research questions concerning how subjective and cognitive measures of cognitive load could reflect load variations are raised.

#### Multimedia and information retention from online news

In spite of growing interest in web user behavior over the last decades, very few studies have addressed the issue of the impact of web design evolution in particular reference to online news. For example, comparing information recall and recognition after reading articles from an online newspaper, Sundar (2000) or Pipps et al. (2009) have shown that content modality impacts user's information retention. Results showed that performance increased when pictures were added to textual news stories whereas the addition of audio or video content led to a drop in performance in information recall. As noted by Sundar (2000) the beneficial effect related to the addition of images can be explained by the dual coding theory (Paivio, 1986) and the cue summation theory (Severin, 1967). The former claims that information is encoded in memory at a verbal and a non-verbal level by means of two independent sub-systems. This theory predicts that a stimulus concurrently presented in both modalities leads to deeper encoding and better recall. Likewise, the cue summation theory posits that images related to the text represent cues for learning and will enhance information retrieval in long-term memory. Regarding the impact of animation, many studies have shown that animations attract user's attention and decrease task performance. For instance, Zhang (2000) indicated that in a web based information search task, animations distracted users and interfered with performance. Specifically, animations such as video advertisements and animated banners are known to be powerful distractor elements (Bayles, 2002; Burke et al., 2004).

Our study dealt with how variations in the amount of multimedia elements affect performance and cognitive load factors. To this end, three versions of an original online newspaper were developed. The general layout was kept constant but the presence of multimedia was modified in each website version (see Appendix 1 for an overview). In the text, pictures and animations version (henceforth referred to as TPA), the articles were presented side by side with images that illustrated the content but did not provide any additional information. These images were of different sizes and positioned alongside the paragraphs. Various animations were added, mainly in the form of animated advertising banners located on the left and right sidebars. A video advertisement was also integrated into the upper middle section at the right of the first paragraph. In the text and pictures version (henceforth referred to as TP)—all animations were removed, and the articles included text and pictures only. The animations zones were replaced by gray tint layers in order to account for the effect of layout. Finally, in the text-only version (henceforth referred to as TO), pictures and animations were removed in order to present a textual version without any multimedia content.

#### Research questions

With respect to CLT principles, the following assumptions can be made. Given that the content of the articles is identical, intrinsic cognitive load (IL)—which is related to the complexity of material to learn—should not differ among the three website versions. In the TPA version, the presence of irrelevant animations will attract the users' attention and place a demand upon cognitive resources and this rise in cognitive load will be extraneous, as it does not contribute to the learning process. Hence, assuming that IL represents a substantial amount of mental resources (Sweller, 2010), the remaining resources available for information processing and encoding—germane load—should be reduced by this rise in extraneous load.

In the original definition of CLT, germane load represents the load devoted to schemata acquisition and automation or, in other words, to learning. Thus, the better the subject's knowledge acquisition from the online stories, the higher the germane load should be. In accordance with this definition, a strong positive relationship between GL and performance is expected. However, as it was argued by certain researchers (Schnotz and Kürschner, 2007; Galy et al., 2012) GL should not be related to task performance but to other cognitive processes such as the application of conscious learning strategies. The germane processes are a function of the cognitive resources available and, furthermore, of the learner's motivation. If GL is effectively related to processes driven by user motivation, one could say that users should have the same motivation to learn in the three different versions of the online newspaper. In order to shed light on the nature of germane load, we therefore propose to examine what are the relationships between GL, performance and cognitive absorption. Derived from the theory of flow (Csikszentmihalyi, 1990), the concept of cognitive absorption is defined as “a state of deep involvement with software” (Agarwal and Karahanna, 2000, p. 673) that encompass the five following dimensions: temporal dissociation (TD), focused immersion (FI), heightened enjoyment (HE), control and curiosity. According to Shang et al. (2005), these dimensions represent different forms of intrinsic motivation. Even though cognitive absorption originally represented a general belief about hypermedia, here it is considered as a measure of immersion in the online newspapers (Jennett et al., 2008). The latest development of subjective rating scales (Leppink et al., 2013) tends to indicate that subjects are able to make the distinction between, and to report, levels of each type of load. We will examine whether subjective measurement can account for the variations in load in the specific context of this study. To the extent that the exact nature of germane load remains unclear for many researchers, relationships with performance and cognitive absorption will be observed.

Finally, in an endeavor to make a contribution to the objective measurement of independent factors of cognitive load, total fixation time in areas of interest will be examined to estimate if user attention has been attracted by the animations in the TPA version. Given that fixation duration increases and saccade length decreases when information processing rises, our study will then observe if there is a difference in these variables among the three newspaper versions when participants are reading the news stories. Finally, a thorough analysis of mean pupil diameter and Index of Cognitive Activity across the whole reading task will be conducted to estimate the total level of cognitive load experienced by users.

## Methods

As stated above, our exploratory study was divided into two phases involving both the same materials and protocol. In the first phase, the main assumptions regarding multimedia influence on performance and variations of cognitive load factors were examined. Relationships between the subjective rating of cognitive loads and cognitive absorption were also observed. In the second phase, eye-tracking data was collected in order to provide reliable and objective measures. According to this two-phase experiment, this section will first present the material and methods used for both phases. Then, an emphasis will be put on the specific methods chosen for the eye-tracking data analysis.

### Material

#### Online newspaper

Three versions of an original online newspaper were developed thanks to the CMS WordPress. We based the design of our website on an existing Belgian online newspaper that has received a number of awards for design. The articles selected for the experiment were inserted into the website's already established layout, resulting in 30 articles brought together in nine categories. The articles were selected on the basis of their relative interest and neutrality and concerned the following topics: Norwegian low cost airlines that had started to offer a service to New York City; The rise of Electronic cigarette usage in Europe, The use of DNA Spray in a burglar alarm. The three websites were hosted on a local server provided by the University in order to get a stable hosting.

#### Measures

Subjective cognitive load was measured by a scale adapted from Leppink et al. (2013) and designed to differentiate the three kinds of load. Taking into account that the scale was developed in the context of a statistics lecture, the items were adjusted to reflect the variations of loads in the specific context of this study. Intrinsic load was evaluated with three items referring to the complexity of the article content (e.g., “The article content was very complex”). Extraneous load with three items related to the supporting effect of website design for learning (e.g., “Website content is not clearly presented”). Germane load was inferred from four items that dealt with the contribution of the reading of articles to knowledge acquisition (e.g., “Reading the article has improved my comprehension of the presented topics”). All items were measured by a 10 point Likert scale ranging from “Strongly disagree” to “Strongly agree.” A factorial analysis performed on the scale across the whole sample (*N* = 128) yielded a perfect three-factor solution. Each dimension showed a good internal consistency with Cronbach's alpha coefficients of 0.827 for IL, 0.701 for EL, and 0.782 for GL. As suggested by Leppink et al. (2013) four items usually used to assess each load in previous studies were added to measure each kind of load. Overall cognitive load was evaluated with an item referring to the overall mental effort invested during the reading (Paas, 1992); IL^2^ with an item referring to the difficulty of the reading (Ayres, 2006); EL^2^ with an item pertaining to the difficulty to learn from the article (Cierniak et al., 2009) and GL^2^ with an item dealing with user's concentration during the reading (Salomon, 1984).

Cognitive absorption was measured by a scale adapted from Agarwal and Karahanna (2000). Although the initial scale contains five dimensions, only three out of five dimensions were measured: FI, TD, and HE^1^. The 11 items were assessed on a 7-point Likert scale ranging from “Strongly disagree” to “Strongly agree.” A three-factor solution was yielded by a factorial analysis and each dimension had a high level of internal consistency (FI: α = 0.862; TD: α = 0.685; HE: 0.868). The users' web expertise and frequency of use of online newspapers were also measured. For the former, participants had to report how much time on average they spent on the internet.

For the task performance measurement, a questionnaire including three recall and three recognition questions were given to the participants for each article. In order to avoid any effect related to the position of information in the paragraphs, the pieces of information to recall were located in paragraphs located at the beginning, the middle and the end of each article. The questions concerned factual information such as quantity or number to ensure that there was no effect from the participants' prior knowledge. The recall questions took the form of sentence gap filling exercises. Subjects had to fill the gap in a sentence from the article with the missing information. In recognition questions, they had to select the right answer from five propositions. One point was granted for each correct answer and zero for an incorrect one. Mean performance scores were calculated for the first two articles in order to avoid serial position effect related to the last one.

### Protocol

The experimental procedure of the first phase was as follows:

- The participants were received in groups of six and randomly assigned to computers running different versions of the experimental website. They were required to fill out a preliminary questionnaire including questions about their age, mother tongue, gender and educational level. Their level of expertise with online newspapers was assessed by the frequency of use of non-specialized online newspapers as well as their Web expertise.
- Participants were asked to read the three articles of the category “Society” by their order of appearance, to wit: The article on the Norwegian airline, that on electronic cigarettes and finally, the DNA spray.
- After reading, participants had to assess the attractiveness, the interest and the complexity of each article and afterwards complete a questionnaire, including the three recall and three recognition questions, for each article.
- Next, participants filled out the cognitive absorption and cognitive load scale. Finally, they were asked to stay in their place until the last person had finished at which point they were invited to attend a debriefing session.

An experimental run took approximately 30 min for each participant. Except that in the second experiment participants were received on a one-to-one basis and the eye-tracker was calibrated, the protocol was strictly adhered to.

### Participants

#### Phase 1

A total of 92 first-year psychology students participated in this study, for which they were given course credit. The participant group consisted of 80 women and 12 men and they had a mean age of 20, with a standard deviation of 3.33. Participants were split among the three newspaper versions (TPA, *N* = 30; TP, *N* = 32; TO, *N* = 30).

#### Phase 2

Thirty-six participants that had not participated in the first study took part in this experiment. They were recruited thanks to a Facebook group dedicated to research experiments and received 7 euros (about 10 $) for their participation. All participants had good vision and did not wear glasses or lens. Mean age was 23 years with a standard deviation of 2.89.

### Apparatus

#### Phase 1

The website was presented in Mozilla Firefox 24 on a 19″ LCD monitor. The computer ran Windows 7 and the resolution was set to 1280 × 1024. Participants were asked to avoid opening new windows or tabs in Mozilla as well as using any other functionality. Mouse and keyboard were the input devices for the interaction.

#### Phase 2

For technical reasons, the website was presented in Internet Explorer 11 instead of Mozilla Firefox. Mouse and keyboard were the input devices for the interaction. The computer ran Windows 7 and the 22″ LCD monitor resolution was set to 1680 × 1050. However, the website was displayed with the same resolution as in Phase 1: 1280 × 1024. Eye-tracking data was recorded using a FaceLab 5 (SeeingMachines™) remote eye-tracking device, sampled at 60 Hz and with an accuracy of 0.5°^2^. The two video cameras and the infra-red spotlight were situated below the screen and participants were asked to stay in the front of cameras during the experimental run. The experiment was conducted in a usability laboratory that allows experimenters to observe and collect data about the users' interactions without interacting with them.

### Eye-tracking data analysis

The software delivered with the eye-tracking bundle (EyeWorks™, EyeTracking Inc.) has been used for extracting raw data and computing the Index of Cognitive Activity. Analyses were then carried out using the open source software OGAMA (Vosskühler et al., 2008). Mean Index of Cognitive Activity and pupil diameter were computed using data from both eyes and minimal fixation duration and spatial area were set to 200 ms and 40 pixels [the expression in pixels of the average system accuracy (1°)] and areas of interest have been drawn around similar sections to distinguish measures for paragraphs, pictures (in TPA and TP versions), peripheral animations and navigation menus. Because of calibration problems and artifacts, 12 participants had been excluded from the eye-tracking data analysis.

## Results

### Phase 1

In order to observe the impact of online newspaper versions in the first experiment (Phase 1), all 10 dependent variables (see Table 1) were first entered together in a One-Way MANOVA with the website version as the independent variable. It revealed a significant multivariate main effect for version [Wilk's λ = 0.465, *F*_(14, 166)_ = 1.917, *p* < 0.01, η^2^ = 0.139]. Given the significance of the overall test, the univariate main effects were examined and described below. Table 1 presents the values (Mean, SD) for the 10 dependent variables in the first phase, respectively for the three versions TPA (text, pictures, animations), TP (text, pictures), and TO (text only).

**Table 1:** **Means (*SD*) of the main dependent variables in the first phase**.

	**Perf**.	**Cognitive load**			**Cognitive absorption**			**Alternatives items for cognitive load**			
		**IL**	**EL**	**GL**	**FI**	**TD**	**HE**	**IL^2^**	**EL^2^**	**GL^2^**	**CL**
TPA *N* = 30	0.52 (0.17)	4.68 (1.61)	4.47 (2.45)	6.40 (1.62)	4.21 (1.19)	4.25 (1.15)	4.13 (1.69)	4.10 (1.32)	5.33 (1.03)	5.23 (1.10)	4.57 (1.19)
TP *N* = 32	0.65 (0.23)	3.95 (1.72)	4.02 (2.01)	6.98 (1.55)	4.67 (1.26)	3.84 (1.09)	4.19 (1.27)	3.94 (1.29)	5.00 (1.34)	5.28 (1.05)	4.66 (1.28)
TO *N* = 30	0.50 (0.18)	4.69 (1.59)	3.80 (1.40)	7.12 (1.33)	3.93 (1.34)	3.93 (1.36)	4.01 (1.30)	4.10 (1.27)	5.23 (1.38)	5.30 (1.47)	4.87 (1.99)

*TD, Temporal dissociation; FI, focused immersion; HE, heightened enjoyment; IL, intrinsic load; EL, extraneous load; GL, germane load; CL, overall cognitive load; IL^2^, intrinsic load alternative item; EL^2^, extraneous load alternative item; GL^2^, germane load alternative item; Perf., performance; TPA, text, pictures, animations; TP, text, pictures; and TO, text without pictures*.

One-Way ANOVA performed on the mean performance scores in Phase 1 yielded a significant main effect [*F*_(2, 91)_ = 5.605, *p* = 0.05, η^2^ = 0.112] of the online newspaper version. A Bonferroni *post-hoc* examination showed that participants' performance was statistically significantly lower in TPA (*M* = 0.52, *SD* = 0.17; *p* < 0.05) and TO (*M* = 0.50; *SD* = 0.18; *p* < 0.01) version compared to the TP version (*M* = 0.65, *SD* = 0.23). There were no statistically significant differences between TPA and TO groups (*p* = 1). Regarding a potential effect of the time taken for reading the articles (see Appendix 2), a Pearson correlation analysis indicated no significant relationship between duration and performance (*r* = 0.119, *p* > 0.05). A One-Way ANOVA confirmed that there were no differences in reading time among the three website versions [*F*_(2, 91)_ = 0.174, *p* > 0.05].

One-Way ANOVAs performed on the three dimensions of subjective rating scale of cognitive load did not find any significant effect of the newspaper version. There was no statistically significant difference in reported IL [*F*_(2, 91)_ = 5.748, *p* = 0.128] but subjects reported a lower level of intrinsic load in the TP version (*M* = 3.95, *SD* = 1.72) than in the TPA (*M* = 4.68, *SD* = 1.61) or TO version (*M* = 4.69, *SD* = 1.59). Despite that, these differences are not significant, Table 1 shows that reported level of extraneous load increased with the presence of animations (see Table 1) while germane load decreased.

When the cognitive absorption scale was subjected to separate One-Way ANOVAs, no significant effects were observed for TD [*F*_(2, 91)_ = 0.99, *p* > 0.05], Enjoyment [*F*_(2, 91)_ = 0.160, *p* > 0.05]. However, FI showed a marginally significant main effect of the website version [*F*_(2, 91)_ = 2.67, *p* = 0.074].

Moreover, a Pearson's correlational analysis was conducted to examine the relationships between cognitive absorption, cognitive load (with the two set of items) and performance for the whole sample (*N* = 128). First, regarding the relationships between loads, correlations (see Table 2) indicated that, as expected, there is a significant negative association between EL and GL (*r* = −470, *p* < 0.01). However, the same pattern was not found between EL^2^ and GL^2^. IL and EL (*r* = 0.277, *p* < 0.01) as well as IL^2^ and EL^2^ (*r* = 0.505, *p* < 0.01) were positively correlated, indicating that the complexity of the material and the complexity of environment were seen as linked by the subjects. Dimensions that related to complexity (IL) or difficulty (IL^2^ and EL^2^) of the content were significantly positively correlated together as well as with the subjective overall cognitive load. IL (*r* = −0.177, *p* < 0.05) and EL^2^ (*r* = −0.314, *p* < 0.01) were also significantly negatively correlated with performance. Put differently, it highlights that the more complex and difficult the articles were perceived to be, the worse was the performance and the greater the reported mental effort. Although a relationship between GL and performance was posited, results revealed that neither GL (*r* = 0.045, *p* > 0.05) nor GL^2^ (*r* = 0.06, *p* > 0.05) were related to performance. Interestingly, only FI showed a significant positive relationship with performance (*r* = 0.237, *p* < 0.01). Conversely, FI was negatively correlated with IL (*r* = −0.196, *p* < 0.05) and EL (*r* = −0.347, *p* < 0.01), showing that subjects reported being less immersed in the reading when the content was perceived as complex and the presentation was seen as unclear. Results underlined the same pattern between FI and IL^2^ (*r* = −0.317, *p* < 0.01) or EL^2^ (*r* = −0.308, *p* < 0.01). Analysis indicated that with both of the two techniques, similar types of load were significantly positively correlated together: IL and IL^2^ (*r* = 0.499, *p* < 0.01), EL and EL^2^ (*r* = 0.374, *p* < 0.01), GL and GL^2^ (*r* = 0.290, *p* < 0.01).

**Table 2:** **Correlations between cognitive load and cognitive absorption dimensions**.

	**Cognitive absorption**			**Cognitive load scale**			**Alternative items for cognitive load**			
	**TD**	**FI**	**HE**	**IL**	**EL**	**GL**	**CL**	**IL^2^**	**EL^2^**	**GL^2^**
TD										
FI	0.460^**^									
HE	0.486^**^	0.465^**^								
IL	0.036	−0.196^*^	−0.004							
EL	−0.133	−0.347^**^	−0.255^**^	0.277^**^						
GL	0.289^**^	0.362^**^	0.401^**^	−0.164	−0.470^**^					
CL	0.065	−0.004	0.193^*^	0.347^**^	0.065	0.0118				
IL^2^	−0.176^*^	−0.317^**^	−0.214^*^	0.499^**^	0.258^**^	−0.238^**^	0.458^**^			
EL^2^	0.154	−0.308^**^	−0.192^*^	0.515^**^	0.374^**^	−0.294^**^	0.308^**^	0.505^**^		
GL^2^	0.066	0.261^**^	0.253^**^	0.152	−0.079	0.290^**^	0.579^**^	0.158	0.143	
Perf.	0.051	0.237^**^	0.114	−0.177^*^	−0.106	0.045	0.077	−0.168	−0.314^**^	–0.006

N = 128;

* p < 0.05;

** *p < 0.01*.

*TD, Temporal dissociation; FI, focused immersion; HE, heightened enjoyment; IL, intrinsic load; EL, extraneous load; GL, germane load; CL, overall cognitive load; IL^2^, intrinsic load alternative item; EL^2^, extraneous load alternative item; GL^2^, germane load alternative item; Perf., performance*.

### Phase 2

Regarding our main dependent variables, results highlighted that the same patterns were found in the two phases (see Appendix 2). Taking into account the very small size of our samples and the supposed violation of normality, only non-parametric test were run to compare the eye-tracking data from the three website versions. However, the results of these tests should be interpreted carefully since it is known that with such a small sample, significance level is almost never reached (Krzywinski and Altman, 2014). Consequently, the main tendencies based on descriptive statistics will be also described below.

To compare the distribution of users' attentional resources between the three website versions, an analysis of the mean number of fixations on each type of design element was conducted. There were more fixations on AOIs related to animations in TPA (*M* = 25.11, *SD* = 29.1) and TO versions (*M* = 25.37, *SD* = 29.27) than in TP version (*M* = 6.57, *SD* = 2.82). The mean total number of fixations on paragraphs showed a smaller increase in TP version (*M* = 484, *SD* = 66.32) than in TPA (*M* = 447.88, *SD* = 57.05) or TO (*M* = 441.62, *SD* = 67.55). In-paragraph pictures were almost equally fixated in TPA (*M* = 23.55, *SD* = 9.18) and TP version (*M* = 29.57, *SD* = 11.47). Menus were fixated on average 4 times (*SD* = 1.87) in TPA, 5 times (*SD* = 2.82) in TP and 8625 times (*SD* = 8.07) in TO. All Kruskal–Wallis^3^ tests ran on total number of fixations on element groups between website versions showed that there were no significant differences.

Means and standard deviations of eye-related dependent variables are presented in Table 3. The first observations (left panel) concern the whole reading task across the three articles, regardless of which specific areas of interest were considered. To get a better understanding of information processing when users were reading the text only, averages have been computed for the paragraphs section (right panel). All Kruskal-Wallis tests ran on these variables did not yield any significant main effect of website version, except for saccade length when computed for paragraphs [*X*^2^(2) = 7.33, *p* < 0.05]. Pairwise comparisons were performed using a Bonferroni correction for multiple comparisons. *Post-hoc* analysis revealed statistically significant differences in saccade length between TO (Mdn = 17.75) and TPA (Mdn = 8.556) (*p* = 0.01) but not between any other combinations.

**Table 3:** **Means (*SD*) of the main dependent eye-related variables for all areas of interest and the paragraphs only**.

	**All AOIs**			**Paragraphs**		
	**TPA**	**TP**	**TO**	**TPA**	**TP**	**TO**
Number of fixation	506.74 (32.21)	537.81 (117.56)	496.13 (81.56)	447.74 (64.26)	483.86 (105.73)	441.63 (94.84)
Fixation	480.05 (82.65)	478.82 (96.5)	462.14 (98.98)	480.46 (91.07)	475.77 (98.63)	461.45 (103.58)
Duration (ms)						
Saccade length (pixels)	141.64 (10.92)	138.86 (4.43)	155.19 (32.72)	121.58 (7.03)	125.12 (3.94)	138.76 (19.39)
Saccade velocity (°/s)	4.52 (0.17)	4.38 (0.27)	4.4 (0.38)	4.43 (0.28)	4.39 (0.28)	4.26 (0.51)
Pupil diameter (mm)	0.00326 (4.395^*^10^−5^)	0.00332 (5.393^*^10^−5^)	0.00324 (5.637^*^10^−5^)	0.00325 (5.21^*^10^−5^)	0.00332 (4.05^*^10^−5^)	0.00323 (5.11^*^10^−5^)
ICA (system unit)	0.363 (0.007)	0.346 (0.020)	0.417 (0.016)	0.363 (0.010)	0.344 (0.019)	0.415 (0.018)

*TPA, text, pictures, animations; TP, text, pictures; and TO, text without pictures*.

**Figure 1:**
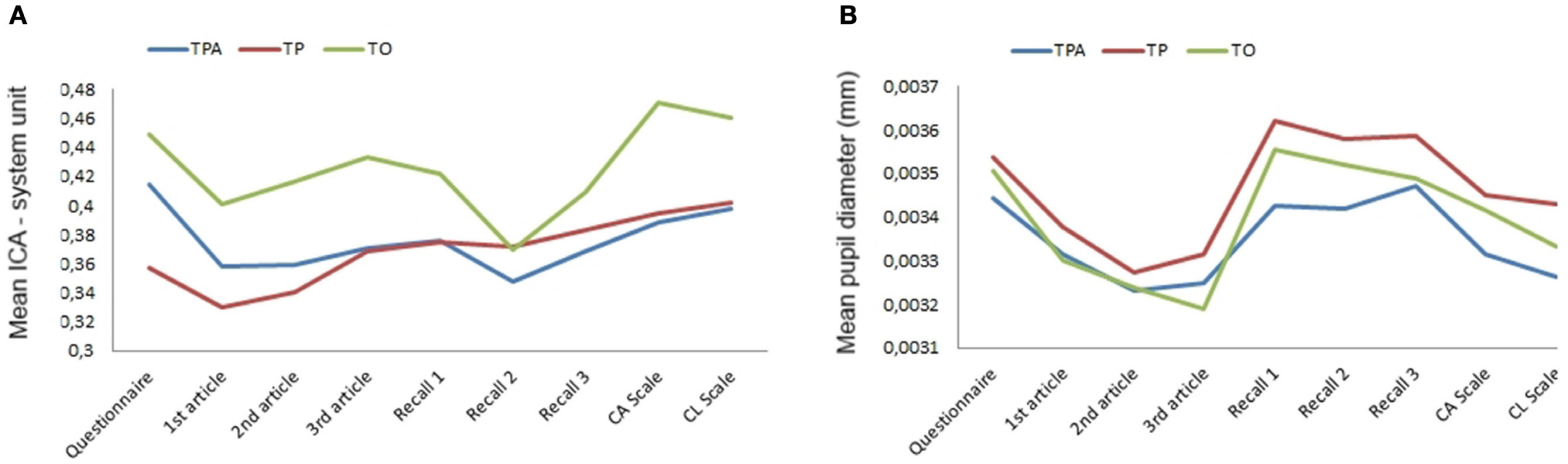
**Phase 2—Mean ICA (A) and mean pupil diameter (B) for each step of the experimental run in text, pictures and animations (TPA); text and pictures (TP), and text-only (TO) version**.

Although these differences are not statistically significant, results (Table 3) indicated that the Index of Cognitive Activity was the highest in TO and the lowest in TP (for both all AOIs and paragraphs only) whereas mean pupil diameter showed an inverted pattern. In an attempt to understand these differences, Friedman tests were conducted to determine if there were any differences in the levels of these two indicators across the different steps of the experimental runs (see Figures 1A,B). Pairwise comparisons were performed with a Bonferroni correction for multiple comparisons between related tasks (completing questionnaires, reading articles, recalling information, filling scales). The mean pupil diameter was statistically significantly different at the different steps during the experimental runs for TPA [*X*^2^(3) = 21.933, *p* < 0.001], TP [*X*^2^(3) = 18.826, *p* < 0.001], and TO [*X*^2^(3) = 21.848, *p* < 0.001]. For every website version, *post-hoc* analysis revealed statistically significant differences in mean pupil diameter from reading articles to recall information. Conversely, the same analysis performed on the Index of Cognitive Activity did not yield any significant differences at the different steps of the experiment, whatever the website version.

**Figure 2:**
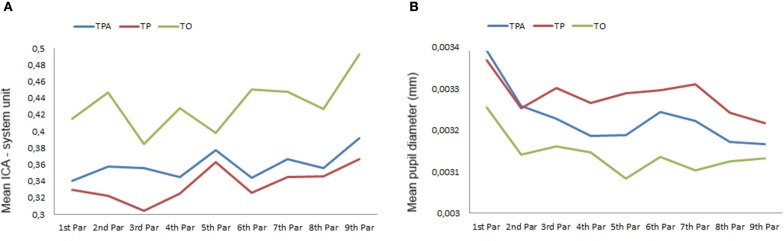
**Phase 2—Mean ICA (A) and mean pupil diameter (B) across each paragraphs read in the second article for text, pictures and animations (TPA); text and pictures (TP), and text-only (TO) version**.

The same analysis was carried out on variations of these two indicators across the paragraphs (see Figures 2A,B) of the second article (to avoid serial position effect). The mean pupil diameter was statistically significantly different across the paragraphs during the reading of the second article for TPA [*X*^2^(3) = 30.933, *p* < 0.001], TP [*X*^2^(3) = 21.998, *p* < 0.01], and TO [*X*^2^(3) = 35.907, *p* < 0.001]. For every website version, *post-hoc* analysis revealed statistically significant differences in mean pupil diameter from the first paragraph to the last one. When performed with the Index of Cognitive Activity data, the Friedman tests yield a significant difference between paragraphs for TPA only [*X*^2^(3) = 16.40, *p* < 0.05] but without any significant pairwise comparison.

## Discussion

The aim of this study was twofold. Firstly, we wanted to investigate whether the CLT framework can be used to explain the impact of multimedia on information retention from online news. Secondly, we wanted to explore to what extent both subjective self-reported scale and eye-related measures can account for the variations of the different types of load described within this frame of reference.

With regard to the first objective, results confirmed that performance in information retention from online news reading was impacted upon by the presence of irrelevant animations. The higher number of fixations on animations in TPA than in TP version suggests that the users' attention could have been attracted by them. Surprisingly, there was as many fixations on animation zones in TO version as in TPA, whereas no animations were present in TO. Even though an artifact related to the eye tracking device accuracy could not be excluded (cf. Limits), it is also possible that user's attention was not well directed by the website layout in TO version. As stated by Sutcliffe and Namoun (2012) allocation of attentional resources in web browsing is driven by top–down mechanisms and bottom–up processes. The former deals with user goals, while the latter is related to layout design and the saliency of the elements. Though the effect of layout design was controlled by replacing animations and pictures with gray layers in TO version, the saliency of these layers may have not been sufficient to guide users' attention. In this case, users may have scanned the page as there were no delimited layout elements and their gazes may have fallen more frequently on the animations zones.

According to CLT framework (Sweller, 1988), we made the assumption that the lower performance in TPA could be explained by a rise of extraneous load in parallel with a decrease of germane load. Unfortunately, our results indicated that both the subjective cognitive load measurement scale and alternative items were unable to discern between specific levels of load among the three website versions. These results are consistent with previous studies that failed to relate differences in performance with variations in types of cognitive load (de Jong, 2010). However, it is noteworthy that we found the expected opposite relationships between EL and GL, showing that when users perceived the content presentation as unclear, they reported a lower contribution to their learning. Even though there were no significant differences in level of IL among website versions, the trend (in both Phase 1 and 2) indicated a lower level of IL in TP version, where the performance was the highest. The negative correlation seems to confirm the opposite relationship between IL and performance and corroborate our previous findings (Debue and Van De Leemput, 2013) since we have previously found that, in web based information search tasks, the reported level of IL was impacted by perceived performance.

Our study also provides interesting findings regarding the nature of germane load. First, the three-factor solution that emerged from the factorial analysis provides some additional support to the triarchic model of cognitive load. Second, in accordance with Leppink et al. (2013), we did not find any relationship between IL and GL (or even IL^2^), which could indicate a non-linear association between these two types of load. As stated by Sweller (2010), GL could not be related to the task characteristics but to learner motivation and may reflect the cognitive resources devoted to dealing with the matter to be learnt. Accordingly, when IL is too low or too high, the subject may not wish to invest further resources in strategies to enhance learning. Interestingly, we found that GL was indeed positively associated with the three dimensions of cognitive absorption, which represent a measure of user motivation (Shang et al., 2005). Thus, when users are intrinsically motivated by the environment they reported a greater ability to devote cognitive resources to learning and their performance improved. These results are consistent with Skadberg and Kimmel's findings (2004) which demonstrated that when users are deeply focused and immersed in a website they tend to learn more about the content presented. Besides the fact that FI is positively linked with performance, there is no correlation between such and GL. This finding seems to provide support to Schnotz and Kürschner's (2007) claim that GL would not be directly related to performance but would represent other cognitive processes that could promote learning.

Taken all together, these observations could explain why the performance was lower in our text-only version (TO) than in text and pictures (TP) version given the absence of distracting animations in both versions. According to Michailidou et al. (2008), aesthetic judgment of web pages is a function of website layout and number of elements such as images and animations present. In this regard, the TO version could have been perceived as less aesthetic and the users may have been less motivated to devote mental resources to retain information from the articles read. This assumption seems to be supported by the lower level of FI reported in TO than in TP version, even if it contrasts with the seductive details theory (Harp and Mayer, 1998) that points out the negative effect of attractive but irrelevant additional content on schemata acquisition and learning. It could be argued that even if seductive details impair the construction of complex schemata by forcing the users to integrate this irrelevant content with essential material (Mayer et al., 2001), this effect only appears when deep learning activities are involved. In the context of simple information retrieval, seductive details seems to enhance user's motivation and task involvement which lead to a better performance in factual information retrieval. Notwithstanding this specific issue, our results are consistent with the cognitive affective theory of learning with media which claims that motivation plays an important role in instigating and maintaining cognitive processing in learning activities (Moreno and Mayer, 2007).

On the whole, these findings indicate that multidimensional subjective measures of cognitive load were able to account for the relationships between cognitive load factors. Nonetheless, variations among the three website versions were not detected which could be due to either a problem of scale sensitivity or to the relativity of the scale. As it has been pointed out by de Jong (2010), we do not know what level of variation is relevant regarding the potential overload that occurs in one condition or in another. One more limitation is that subjective scales cannot account for variations over time but reflect only the average load experienced by the subject over the whole learning task. Hence, our study investigated whether objective measures could account for these variations as well as whether they can be related to specific types of load.

Despite our small sample size and the lack of statistical significance, the trends observed in our exploratory eye-tracking experiment provide some interesting findings. Longer fixations and shorter saccades were found in TPA version, which could indicate that (extraneous) cognitive load was higher and that more attentional resources were required to read the articles when subjects faced distracting animations. This result is in line with findings from several studies that highlighted the relationship between higher cognitive load and longer fixation (De Greef et al., 2009; Liu et al., 2011; Buettner, 2013) and shorter saccade (Chen et al., 2011; Holmqvist et al., 2011).

Interestingly, our trends indicated that the mean pupil diameter was higher in TP whereas it was equivalent in the two other versions. It could be underlined that variations were very small but such as small difference have been found as significant in other studies (De Greef et al., 2009). As pupillary response has been associated with information processing (Backs and Walrath, 1992) it is noteworthy that pupil diameter was higher only in the version which had led to the best performance. Nevertheless, our results cast some doubts on the nature of processes which are reflected by the variations of pupil diameter. Although the pupil variations observed across the different steps of the experiment could be explained by the luminance variation, the decrease in this indicator while subjects went along the paragraphs is rather astonishing. Furthermore, while both mean pupil diameter and the Index of Cognitive Activity were supposed to be related to cognitive demand, we found that they did not show the same patterns of variation, which raises many issues about the exact nature of these measures.

Our study suffers from some limitations that should be taken into consideration. First, our sample size in the second phase of our experiment was very limited. Even though our eye-tracking results showed relevant trends, a larger sample size would have provided a better statistical power. Second, regarding our measurement tools, the subjective scale we used to measure cognitive load was adapted from a very different situation (a statistics lecture). Even if the items were modified in order to fit into our research context, a scale specifically designed for evaluating cognitive load in the use of hypermedia might have provided more accurate and reliable results. Moreover, we think that a more sensitive scale, ranging from 1 to 100 instead of a 10 point Likert scale for instance, would have been able to detect subtler variations of load among the website versions. In addition, we administered the scale at the end of the task and, in this way, our subjective cognitive load rating could not take into account the variations of load over time. It would have been interesting to present the scale after the end of each article in order to overcome these limitations and to provide a better temporal accuracy. Concerning the eye-related measures, it should be noted that the definition of the areas of interest and the relative accuracy of the eye-tracking device may have produced some artifacts with regards to measures that were computed for these areas. Third, we could also formulate some remarks about the design used in this study. While a three-condition design has provided many interesting results that would not have emerged with only two conditions, it had increased the complexity of the analysis and a two-condition design would have been better in terms of statistical power. On another note, because the eye-related measures vary greatly from one individual to another—they are *idiosyncratic*—it might have been difficult to distinguish the variations between participants from those caused by the differences among website versions. A within-subjects design, where participants would have to go through each version and the presentation order would be counterbalanced, should be applied in further experiments.

In conclusion, it appears from the aforementioned findings that cognitive load is a multidimensional concept that encompasses several factors. By providing some understandings of the means that can be used to gauge the cognitive load, our study sets the stage for further confirmatory studies.

### Conflict of interest statement

The authors declare that the research was conducted in the absence of any commercial or financial relationships that could be construed as a potential conflict of interest.

## Footnotes

^1^Because subjects were explicitly asked to use the online newspaper during the experiment, control and curiosity were not retained in the definition of CA.

^2^According to Bartels and Marshall (2012), the Index of Cognitive Activity is not influenced by the sampling rate and can be registered with slow rate (60 Hz) eye tracking devices.

^3^A Mann–Whitney test was run for in-paragraph pictures.

## References

[B1] Agarwal R. Karahanna E. (2000). Time flies when you're having fun: cognitive absorption and beliefs about information technology usage. MIS Q. 24, 665–694 10.2307/3250951

[B2] Albers M. J. (2011). Tapping as a measure of cognitive load and website usability, in Proceeding SIGDOC '11 Proceedings of the 29th ACM International Conference on Design of Communication (New York, NY: ACM), 25–32 10.1145/2038476.2038481

[B3] Amadieu F. Tricot A. (2006). Utilisation d'un hypermédia et apprentissage: deux activités concurrentes ou complémentaires? Psychol. Fr. 51, 5–23 10.1016/j.psfr.2005.12.001

[B4] Amadieu F. van Gog T. Paas F. Tricot A. Mariné C. (2009). Effects of prior knowledge and concept-map structure on disorientation, cognitive load, and learning. Learn. Instr. 19, 376–386 10.1016/j.learninstruc.2009.02.005

[B5] Ayaz H. Shewokis P. A. Bunce S. Izzetoglu K. Willems B. Onaral B. (2012). Optical brain monitoring for operator training and mental workload assessment. Neuroimage 59, 36–47 10.1016/j.neuroimage.2011.06.023 21722738

[B6] Ayres P. (2006). Using subjective measures to detect variations of intrinsic cognitive load within problems. Learn. Instr. 16, 389–400 10.1016/j.learninstruc.2006.09.001

[B7] Backs R. W. (1995). Going beyond heart rate: autonomic space and cardiovascular assessment of mental workload. Int. J. Aviat. Psychol. 5, 25–48 10.1207/s15327108ijap0501_3 11541494

[B8] Backs R. W. Walrath L. C. (1992). Eye movement and pupilary response indices of mental workload during visual search of symbolic displays. Appl. Ergon. 23, 243–254 10.1016/0003-6870(92)90152-L 15676872

[B8a] Bartels M. Marshall S. (2012). Measuring cognitive workload across different eye tracking hardware platforms, in Proceedings of 2012 Eye Tracking Research and Applications Symposium (New York, NY: ACM), 161–164 10.1145/2168556.2168582

[B9] Bayles M. E. (2002). Designing online banner advertisements: should we animate? in Proceedings of the SIGCHI Conference on Human Factors in Computing Systems CHI'02 (New York, NY: ACM), 363–366

[B10] Brunken R. Plass J. L. Leutner D. (2003). Direct measurement of cognitive load in multimedia learning. Educ. Psychol. 38, 53–61 10.1207/S15326985EP3801_7 12053529

[B11] Buettner R. (2013). Cognitive workload of humans using artificial intelligence systems: towards objective measurement applying eye-tracking technology, in KI 2013: Advances in Artificial Intelligence, Lecture Notes in Computer Science, eds Timm I. J. Thimm M. (Berlin: Springer), 37–48 10.1007/978-3-642-40942-4-4

[B12] Burke M. Gorman N. Nilsen E. Hornof A. (2004). Banner ads hinder visual search and are forgotten, in CHI'04 Extended Abstracts on Human Factors in Computing Systems CHI EA'04 (New York, NY: ACM), 1139–1142 10.1145/985921.986008

[B13] Ceggara J. Chevalier A. (2008). The use of Tholos software for combining measures of mental workload: toward theoretical and methodological improvements. Behav. Res. Methods 40, 988–1000 10.3758/brm.40.4.988 19001390

[B14] Chanquoy L. Tricot A. Sweller J. (2007). La Charge Cognitive: Théories et Applications. Paris: Armand Colin

[B15] Chen S. Epps J. (2012). Automatic classification of eye activity for cognitive load measurement with emotion interference. Comput. Methods Programs Biomed. 110, 111–124 10.1016/j.cmpb.2012.10.021 23270963

[B16] Chen S. Epps J. Ruiz N. Chen F. (2011). Eye activity as a measure of human mental effort in HCI in Proceeding IUI '11 Proceedings of the 16th International Conference on Intelligent User Interfaces (New York, NY: ACM), 315–318 10.1145/1943403.1943454

[B17] Chevalier A. Kicka M. (2006). Web designers and web users: influence of the ergonomic quality of the web site on the information search. Int. J. Hum.-Comput. Stud. 64, 1031–1048 10.1016/j.ijhcs.2006.06.002

[B18] Chi M. T. Glaser R. Rees E. (1981). Expertise in Problem Solving. Pittsburgh, PA: Learning Research and Development Center, University of Pittsburgh

[B19] Cierniak G. Scheiter K. Gerjets P. (2009). Explaining the split-attention effect: is the reduction of extraneous cognitive load accompanied by an increase in germane cognitive load? Comput. Hum. Behav. 25, 315–324 10.1016/j.chb.2008.12.020

[B20] Csikszentmihalyi M. (1990). Flow: the Psychology of Optimal Experience. New York, NY: Harper & Row

[B21] Debue N. Van De Leemput C. (2013). Acceptabilité des sites web et ergonomie de l'interface: étude de l'influence de l'utilisabilité objective et de la charge cognitive. Rev. Interact. Hum. Médiatisées 14, 1–23

[B22] De Greef T. Lafeber H. van Oostendorp H. Lindenberg J. (2009). Eye movement as indicators of mental workload to trigger adaptive automation, in Foundations of Augmented Cognition. Neuroergonomics and Operational Neuroscience Lecture Notes in Computer Science, eds Schmorrow D. D. Estabrooke I. V. Grootjen M. (Berlin: Springer), 219–228

[B23] de Jong T. (2010). Cognitive load theory, educational research, and instructional design: some food for thought. Instr. Sci. 38, 105–134 10.1007/s11251-009-9110-0

[B24] DeLeeuw K. E. Mayer R. E. (2008). A comparison of three measures of cognitive load: evidence for separable measures of intrinsic, extraneous, and germane load. J. Educ. Psychol. 100, 223–234 10.1037/0022-0663.100.1.223

[B25] DiStasi L. L. Antolí A. Gea M. Cañas J. J. (2011). A neuroergonomic approach to evaluating mental workload in hypermedia interactions. Int. J. Ind. Ergon. 41, 298–304 10.1016/j.ergon.2011.02.008

[B26] Galy E. Cariou M. Mélan C. (2012). What is the relationship between mental workload factors and cognitive load types? Int. J. Psychophysiol. 83, 269–275 10.1016/j.ijpsycho.2011.09.023 22008523

[B27] Gerjets P. Scheiter K. Catrambone R. (2004). Designing instructional examples to reduce intrinsic cognitive load: molar versus modular presentation of solution procedures. Instr. Sci. 32, 33–58 10.1023/b:truc.0000021809.10236.71

[B28] Gerjets P. Scheiter K. Cierniak G. (2009). The scientific value of cognitive load theory: a research Agenda based on the structuralist view of theories. Educ. Psychol. Rev. 21, 43–54 10.1007/s10648-008-9096-1

[B29] Harp S. F. Mayer R. E. (1998). How seductive details do their damage: a theory of cognitive interest in science learning. J. Educ. Psychol. 90, 414–434 10.1037/0022-0663.90.3.414

[B30] Hart S. G. Staveland L. E. (1988). Development of NASA-TLX (Task Load Index): results of empirical and theoretical research. Adv. Psychol. 52, 139–183 10.1016/S0166-4115(08)62386-9

[B31] Hitch G. J. Baddeley A. D. (1976). Verbal reasoning and working memory. Q. J. Exp. Psychol. 28, 603–621 10.1080/14640747608400587

[B32] Hollender N. Hofmann C. Deneke M. Schmitz B. (2010). Integrating cognitive load theory and concepts of human-computer interaction. Comput. Hum. Behav. 26, 1278–1288 10.1016/j.chb.2010.05.031

[B33] Holmqvist K. Nyström M. Andersson R. Dewhurst R. Jarodzka H. van de Weijer J. (2011). Eye Tracking: A Comprehensive Guide To Methods And Measures. New York, NY: Oxford University Press

[B34] Hyönä J. (2010). The use of eye movements in the study of multimedia learning. Learn. Instr. 20, 172–176 10.1016/j.learninstruc.2009.02.013

[B35] Jainta S. Baccino T. (2010). Analyzing the pupil response due to increased cognitive demand: an independent component analysis study. Int. J. Psychophysiol. 77, 1–7 10.1016/j.ijpsycho.2010.03.008 20381549

[B36] Jennett C. Cox A. L. Cairns P. Dhoparee S. Epps A. Tijs T. (2008). Measuring and defining the experience of immersion in games. Int. J. Hum.-Comput. Stud. 66, 641–661 10.1016/j.ijhcs.2008.04.004

[B37] Kalyuga S. (2011). Cognitive load theory: how many types of load does it really need? Educ. Psychol. Rev. 23, 1–19 10.1007/s10648-010-9150-7

[B38] Kirschner P. A. Ayres P. Chandler P. (2011). Contemporary cognitive load theory research: the good, the bad and the ugly. Comput. Hum. Behav. 27, 99–105 10.1016/j.chb.2010.06.025

[B39] Krzywinski M. Altman N. (2014). Points of significance: nonparametric tests. Nat. Methods 11, 467–468 10.1038/nmeth.2937 24820360

[B40] Leppink J. Paas F. Vleuten C. P. M. V. der Gog T. V. Merriënboer J. J. G. V. (2013). Development of an instrument for measuring different types of cognitive load. Behav. Res. Methods 45, 1058–1072 10.3758/s13428-013-0334-1 23572251

[B41] Liu H.-C. Lai M.-L. Chuang H.-H. (2011). Using eye-tracking technology to investigate the redundant effect of multimedia web pages on viewers' cognitive processes. Comput. Hum. Behav. 27, 2410–2417 10.1016/j.chb.2011.06.012

[B42] Marshall S. P. (2000). Method and apparatus for eye tracking and monitoring pupil dilation to evaluate cognitive activity. U.S. Patent No 6,090,051. Available online at: http://ingentaconnect.com/content/asma/asem Washington, DC: U.S. Patent and Trademark Office

[B43] Marshall S. P. (2002). The index of cognitive activity: measuring cognitive workload, in Proceedings of the 2002 IEEE 7th Conference on Human Factors and Power Plants, 2002 (New York, NY: IEEE), 7–5–7–9.

[B44] Marshall S. P. (2007). Identifying cognitive state from eye metrics. Aviat. Space Environ. Med. 78, B165–B175 17547317

[B45] Mayer R. E. Heiser J. Lonn S. (2001). Cognitive constraints on multimedia learning: when presenting more material results in less understanding. J. Educ. Psychol. 93, 187–198 10.1037/0022-0663.93.1.187

[B46] Mehler B. Reimer B. Coughlin J. F. Dusek J. A. (2009). Impact of incremental increases in cognitive workload on physiological arousal and performance in young adult drivers. Transp. Res. Rec. J. Transp. Res. Board 2138, 6–12 10.3141/2138-02

[B47] Michailidou E. Harper S. Bechhofer S. (2008). Visual complexity and aesthetic perception of web pages, in Proceedings of the 26th Annual ACM International Conference on Design of Communication SIGDOC'08 (New York, NY: ACM), 215–224 10.1145/1456536.1456581

[B48] Moreno R. Mayer R. (2007). Interactive multimodal learning environments. Educ. Psychol. Rev. 19, 309–326 10.1007/s10648-007-9047-2

[B49] Mulder G. Mulder L. J. M. (1981). Information processing and cardiovascular control. Psychophysiology 18, 392–402 10.1111/j.1469-8986.1981.tb02470.x 7267921

[B50] Paas F. G. W. C. (1992). Training strategies for attaining transfer of problem-solving skill in statistics: a cognitive-load approach. J. Educ. Psychol. 84, 429–434 10.1037/0022-0663.84.4.429

[B51] Paas F. Tuovinen J. E. Tabbers H. Van Gerven P. W. M. (2003). Cognitive load measurement as a means to advance cognitive load theory. Educ. Psychol. 38, 63–71 10.1207/S15326985EP3801_8

[B52] Paas F. Van Merriënboer J. (1994). Instructional control of cognitive load in the training of complex cognitive tasks. Educ. Psychol. Rev. 6, 351–371 10.1007/bf0221342023968802

[B53] Paivio A. (1986). Mental Representations: A Dual Coding Approach. New York, NY: Oxford University Press

[B54] Pipps V. Walter H. Endres K. Tabatcher P. (2009). Information recall of internet news: does design make a difference? A pilot study. J. Mag. New Media Res. 11, 1–20

[B55] Rayner K. (1998). Eye movements in reading and information processing: 20 years of research. Psychol. Bull. 124, 372–422 10.1037/0033-2909.124.3.372 9849112

[B56] Salomon G. (1984). Television is “easy” and print is “tough”: the differential investment of mental effort in learning as a function of perceptions and attributions. J. Educ. Psychol. 76, 647–658 10.1037/0022-0663.76.4.647

[B57] Scheiter K. Gerjets P. Catrambone R. (2006). Making the abstract concrete: visualizing mathematical solution procedures. Comput. Hum. Behav. 22, 9–25 10.1016/j.chb.2005.01.009

[B58] Schnotz W. Kürschner C. (2007). A reconsideration of cognitive load theory. Educ. Psychol. Rev. 19, 469–508 10.1007/s10648-007-9053-4

[B59] Severin W. (1967). Another look at cue summation. AV Commun. Rev. 15, 233–245

[B60] Shang R.-A. Chen Y.-C. Shen L. (2005). Extrinsic versus intrinsic motivations for consumers to shop on-line. Inf. Manag. 42, 401–413 10.1016/j.im.2004.01.009

[B61] Skadberg Y. X. Kimmel J. R. (2004). Visitors' flow experience while browsing a web site: its measurement, contributing factors and consequences. Comput. Hum. Behav. 20, 403–422 10.1016/S0747-5632(03)00050-5

[B62] Sundar S. S. (2000). Multimedia effects on processing and perception of online news: a study of picture, audio, and video downloads. Journal. Mass Commun. Q. 77, 480–499 10.1177/107769900007700302

[B63] Sutcliffe A. Namoun A. (2012). Predicting user attention in complex web pages. Behav. Inf. Technol. 31, 679–695 10.1080/0144929X.2012.692101

[B64] Sweller J. (1988). Cognitive load during problem solving - effects on learning. Cogn. Sci. 12, 257–285 10.1207/s15516709cog1202_4

[B65] Sweller J. (2010). Element interactivity and intrinsic, extraneous, and germane cognitive load. Educ. Psychol. Rev. 22, 123–138 10.1007/s10648-010-9128-524593808

[B66] Sweller J. van Merrienboer J. J. G. Paas F. G. W. C. (1998). Cognitive architecture and instructional design. Educ. Psychol. Rev. 10, 251–296 10.1023/A:1022193728205

[B67] VanGerven P. W. M. Paas F. Van Merriënboer J. J. G. Schmidt H. G. (2004). Memory load and the cognitive pupillary response in aging. Psychophysiology 41, 167–174 10.1111/j.1469-8986.2003.00148.x 15032982

[B68] VanGog T. Kester L. Nievelstein F. Giesbers B. Paas F. (2009). Uncovering cognitive processes: different techniques that can contribute to cognitive load research and instruction. Comput. Hum. Behav. 25, 325–331 10.1016/j.chb.2008.12.021

[B69] Vosskühler A. Nordmeier V. Kuchinke L. Jacobs A. M. (2008). OGAMA (Open Gaze and Mouse Analyzer): open-source software designed to analyze eye and mouse movements in slideshow study designs. Behav. Res. Methods 40, 1150–1162 10.3758/BRM.40.4.1150 19001407

[B70] Whelan R. R. (2007). Neuroimaging of cognitive load in instructional multimedia. Educ. Res. Rev. 2, 1–12 10.1016/j.edurev.2006.11.001

[B71] Zhang P. (2000). The effects of animation on information seeking performance on the world wide web: securing attention or interfering with primary tasks? J. AIS. 1, 1–28

