# Supplementary material for: What does germane load mean? An empirical contribution to the cognitive load theory
Source: Front Psychol. 2014 Oct 1;5:1099. doi: 10.3389/fpsyg.2014.01099 (PMC4181236; doi:10.3389/fpsyg.2014.01099)
Supplement: Supplementary file 1 [file Presentation1.PDF]

7. Appendix

Appendix 1. Screenshots of the three versions (from left to right: TPA, TP and TO) of the prototypical online newspaper

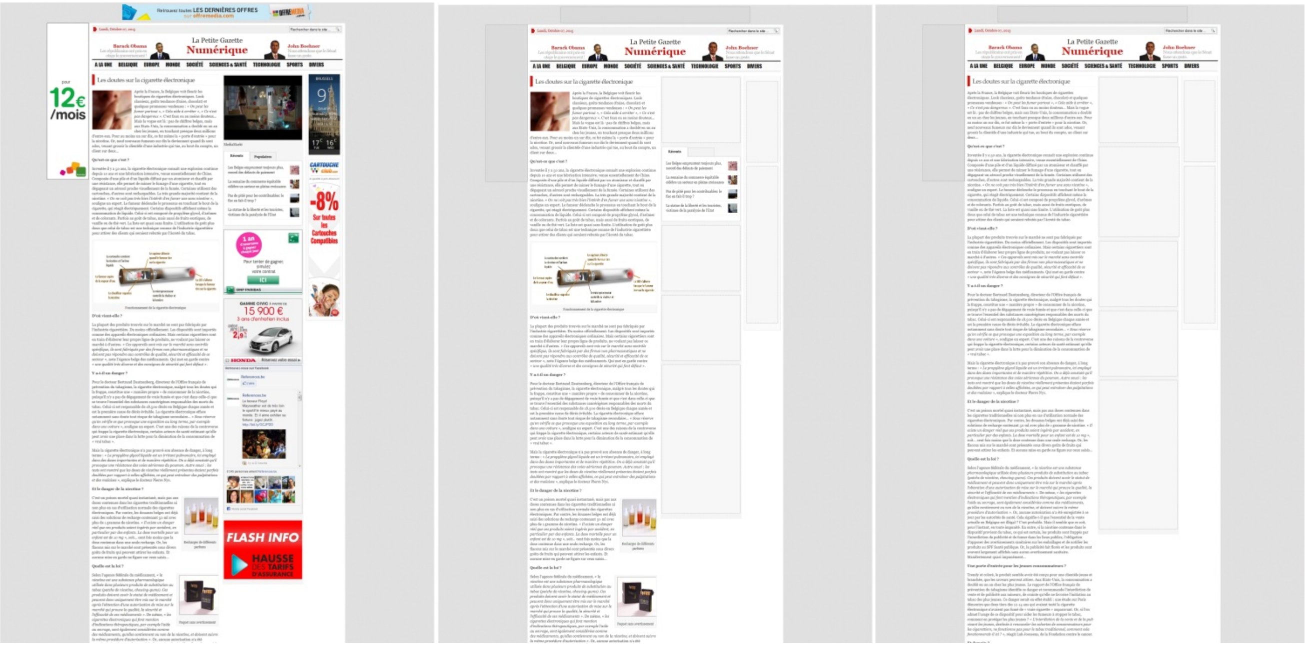

Appendix 2. Means (*SD*) of the main dependent variables studied in both the first (P1) and the second phase (P2) among the text, pictures and animations (TPA); text and pictures (TP) and text-only (TO) version

**Variables**	**TPA**		**TP**		**TO**		**TOTAL**	
Phase	**P1**	**P2**	**P1**	**P2**	**P1**	**P2**	**P1**	**P2**
N	30	13	32	11	30	12	92	36
Time	1156.77	1047.53	1117.22	1002.36	1097.53	917.41	1123.70	990.36
	(581.67)	*(315.86)*	*(247.65)*	*(261.29)*	*(51.19)*	*(191.11)*	*(392.90)*	*(261.29)*
Web expertise	4.77	3.85	3.66	3.82	4.57	4.25	4.32	3.97
	(2.64)	*(1.72)*	*(1.60)*	*(1.33)*	*(2.63)*	*(2.01)*	*(2.35)*	*(1.25)*
Frequency of use	2.77	3.38	2.78	3.27	2.30	3.58	2.62	3.42
	(1.30)	*(1.45)*	*(1.41)*	*(1.10)*	*(1.44)*	*(1.24)*	*(1.91)*	*(1.25)*
***Cognitive load* scale (Leppink et al.. 2013)**								
Intrinsic Load	4.68	4.46	3.95	3.70	4.69	4.65	4.43	4.29
	(1.61)	*(1.68)*	*(1.72)*	*(1.46)*	*(1.59)*	*(1.45)*	*(1.66)*	*(1.55)*
Extraneous Load	4.47	4.49	4.02	2.74	3.80	3.89	4.09	4.04
	(2.45)	*(1.97)*	*(2.01)*	*(1.38)*	*(1.40)*	*(1.85)*	*(2.00)*	*(1.75)*
Germane Load	6.40	7.19	6.98	7.32	7.12	7.52	6.84	7.34
	(1.62)	*(1.44)*	*(1.55)*	*(1.42)*	*(1.33)*	*(1.26)*	*(1.52)*	*(1.34)*
Cognitive absorption (Agarwal & Karahanna. 2000)								
Focused	4.21	4.48	4.67	4.56	3.93	4.25	4.28	3.89
Immersion	*(1.19)*	*(1.24)*	*(1.26)*	*(1.12)*	*(1.34)*	*(1.17)*	*(1.29)*	*(1.13)*
Temporal	4.25	3.72	3.84	4.03	3.93	3.94	4.01	4.43
Dissociation	*(1.15)*	*(1.41)*	*(1.09)*	*(1.16)*	*(1.36)*	*(0.80)*	*(1.20)*	*(1.15)*
Heightened	4.13	4.38	4.19	4.68	4.01	3.98	4.11	4.34
Enjoyment	*(1.69)*	*1(.26)*	*(1.27)*	*(1.26)*	*(1.30)*	*(1.10)*	*(1.31)*	*(1.21)*
Alternative items for subjective cognitive load measurement								
Overall Load	4.57	4.54	4.66	5.09	4.87	4.92	4.70	4.83
	(1.19)	*(1.12)*	*(1.28)*	*(1.57)*	*(1.99)*	*(1.08)*	*(1.51)*	*(1.25)*
Intrinsic Load	4.10	4.00	3.94	4.00	4.10	3.92	4.04	3.97
	(1.32)	*(1.29)*	*(1.29)*	*(1.61)*	*(1.27)*	*(.90)*	*(1.28)*	*(1.25)*
Extraneous Load	5.33	5.23	5.00	4.73	5.23	5.50	5.18	5.17
	(1.02)	*(1.17)*	*(1.34)*	*(1.74)*	*(1.38)*	*(.80)*	*(1.26)*	*(1.28)*
Germane Load	5.23	5.54	5.28	5.82	5.30	5.50	5.27	5.61
	(1.10)	*(1.39)*	*(1.05)*	*(1.08)*	*(1.47)*	*(1.17)*	*(1.20)*	*(1.20)*
